# The Blind Soldier

**Published:** 1915-11-27

**Authors:** 


### THE BLIND SOLDIER.

Upon the outbreak of the war it was realised that there was a great need for an institution which could receive, care for, and train those who might lose their power of sight while on service for their country. As a result the Hostel of St. Dunstan's, Regent's Park, was established, and is being maintained by the National Institute for the Blind, the British Red Cross Society, and the Order of St. John of Jerusalem for the purpose; the management is in the hands of the Blinded Soldiers and Sailors Care Committee.

The hostel, which was offered by Mr. Otto Kahn, is a building very admirably suited for the patients, standing in some fifteen acres of ground; it includes a portion of the Regent's Park lake, which affords facilities for rowing—a pastime which is keenly appreciated by the blind inmates.

The men come from the various military hospitals, and are afforded the opportunity at St. Dunstan's of learning a trade by which they may reasonably hope, after a period of training averaging about six months, to earn a livelihood for themselves and for any who may be dependent upon them. The occupations taught are many and varied; instruction is given in carpentry, boot repairing, mat and basket making, massage, and telephony; some two and a half hours each day are devoted to this branch of work, while a similar period is spent in learning Braille reading, writing, and typewriting.

Perhaps one of the most interesting adjuncts to this merciful institution is the model farm, under the care of Captain Webber, one of the greatest blind experts in the country. In this country-life section the men are taught market gardening, beekeeping, and poultry farming; and by clever devices it is possible for the patients to carry on the work with great success. After a short period of training they can drive birds from one pen to another, catch any particular fowl, and collect eggs.

As already mentioned, rowing is a favourite recreation. Arrangements have also been made for attendance at the St. Marylebone Baths at certain times on certain days of the week, and it is stated that some of those who were non-swimmers on their coming to the hostel are rapidly becoming experts in the art. The Council of the National Institute for the Blind has placed at the disposal of the committee its seaside home at Brighton, where men can be sent to recuperate after discharge from hospital and previous to their taking up their training at St. Dunstan's.

The staff is drawn to a large extent from members of various Voluntary Aid Detachments, and the instructors are largely voluntary. Perhaps the real secret of the success of this happy institution is the spirit of cheerful optimism and hope which prevails throughout. The Secretary of State for War has approved of the arrangements which have been made for providing additional accommodation at the Blinded Soldiers' Hostel to an extent which will enable 120 men to be cared for and trained there.

